# A *Mycobacterium bovis* BCG-Naked DNA Prime-Boost Vaccination Strategy Induced CD4^+^ and CD8^+^ T-Cell Response against *Mycobacterium tuberculosis* Immunogens

**DOI:** 10.1155/2014/395626

**Published:** 2014-03-11

**Authors:** Miao Lu, Zhi Yang Xia, Lang Bao

**Affiliations:** Laboratory of Infection and Immunity, School of Basic Medical Science, West China Center of Medical Sciences, Sichuan University, No. 17, Third Section, Ren Min Nan Road, Chengdu, Sichuan 610041, China

## Abstract

*Mycobacterium tuberculosis* infection is still a major global public health problem. Presently the only tuberculosis (TB) vaccine available is Bacille Calmette-Guérin (BCG), although it fails to adequately protect against pulmonary TB in adults. To solve this problem, the development of a new effective vaccine is urgently desired. BCG-prime DNA-booster vaccinations strategy has been shown to induce greater protection against tuberculosis (TB) than BCG alone. Some studies have demonstrated that the two genes (Rv1769 and Rv1772) are excellent T-cell antigens and could induce T-cell immune responses. In this research, we built BCG-C or BCG-P prime-recombination plasmid PcDNA3.1-Rv1769 or PcDNA3.1-Rv1772 boost vaccinations strategy to immunize BALB/c mice and evaluated its immunogenicity. The data suggests that the BCG-C+3.1-72 strategy could elicit the most long-lasting and strongest Th1-type cellular immune responses and the BCG-C+3.1-69 strategy could induce the high level CD8+ T-cell response at certain time points. These findings support the ideas that the prime-boost strategy as a combination of vaccines may be better than a single vaccine for protection against tuberculosis.

## 1. Introduction

Today tuberculosis (TB) still remains a major infectious cause of morbidity and mortality worldwide, one-third of the world's population is latently infected with* Mycobacterium tuberculosis*. Bacille Calmette-Guérin (BCG) is the only available vaccine against tuberculosis presently, and the protective efficacy of it is variable from 0 to 80% in many field trials and is unclear in pulmonary TB in adults [1], but it prevents miliary tuberculosis in newborns and toddlers [2, 3]. The cause of these large differences in vaccine-induced protection is poorly understood, and some of its limitations may involve short-lived BCG-induced immune reactivity. This raises an issue that a novel TB vaccine which can protect adults against tuberculosis is urgently needed [3, 4], while BCG vaccination of newborns should be continued as it is effective. Previous TB vaccines are classified into 4 main groups: (1) DNA vaccines, (2) recombinant BCG vaccines, (3) subunit vaccines, and (4) attenuated vaccines, and currently most efforts to improve the protective immunity of BCG are focused on strategies that incorporate priming with BCG, recombinant BCG, or other attenuated mycobacteria followed by a heterologous booster immunization that aims to improve the duration and efficacy of the responses [5–7]. Considering all kinds of reasons, we decide to choose the heterologous prime-boost vaccination strategy comprised of priming with BCG and boosting with a novel vaccine candidate [8, 9].

Cellular immune responses are critical for the control of* Mycobacterium tuberculosis* infection, which depends on polyfunctional CD4^+^ and CD8^+^ T-cell responses [10, 11]. T helper type 1 (Th1) CD4^+^ T cell can primarily secrete interferon-*γ* (IFN-*γ*), which is important in* M. tuberculosis *infection and disease prevention [12]. CD8^+^ cytolytic T lymphocytes (CTLs) are essential for clearance of intracellular* M. tuberculosis* infection [13] by secreting perforin, granulysin, and extracellular enzymes into the immunological synapse [14]. Heterologous prime-boost strategy has been used in many models of pathogenic infections [15], and some studies demonstrate that prime-boost strategies using BCG as prime and heterologous constructs such as recombinant DNA, recombinant adenovirus, and recombinant poxviruses as boosting immunogens can enhance CD4^+^ and CD8^+^ T-cell responses against TB [6, 7, 16–18].

To search for a novel effective vaccine candidate to improve the protection of BCG, many strategies have been attempted and a number of antigens have been studied. In our research, we choose two BCG substrains (BCG-Pasteur1173 and BCG-China) which are different in two deletions called RD14 and N-RD18 [19, 20], which are present in BCG-China, but absent in BCG-Pasteur1173. We notice two genes (Rv1769 and Rv1772) in RD14 deletion, which have been studied superficially, and some research has indicated that Rv1769 and Rv1772 should be considered for potential subunit vaccines [21, 22].

In previous work, researchers paid much attention to ESAT-6, CFP-10, and Ag85 [7, 16, 23–25], and little attention has been paid to the RD14 deletion. Maybe the genes located in this deletion are responsible for different immunogenicity between the BCG-Pasteur and BCG-China. Based on all of the reasons above, we have constructed several vaccination strategies primed with BCG-C or BCG-P and boosted with recombination plasmid pcDNA3.1-Rv1769 or pcDNA3.1-Rv1772 to immunize BALB/c mice and evaluated its immunogenicity. This study shows that this strategy can elicit potent humoral and cellular immune responses comprising both CD4^+^ and CD8^+^ T cells against TB in mice, but its protective efficacy was not to be demonstrated in this study.

## 2. Materials and Methods

### 2.1. Bacterial Strains, Media, and Plasmids

*M. bovis *BCG-Pasteur and BCG-China were kindly provided by the Chengdu Biological Products Institute. BCGs were maintained in Sauton's medium (MgSO_4_ 0.5 g, K_2_HPO_4_ 0.5 g, citric acid 2 g, sodium glutamate 8 g, glycerol 60 mL, ZnSO_4_ 0.01 g, and ferrum-ammonium citrate 0.05 g in 1000 mL, pH1.4–7.5). The plasmid was originally conserved in our laboratory.

### 2.2. Plasmid Construction

The Rv1769 and Rv1772 genes were amplified from the BCG-China genome and cloned into pcDNA3.1(+) plasmid to generate recombinant pcDNA3.1-Rv1769 and pcDNA3.1-Rv1772. The sequences were confirmed by sequencing by Invitrogen (Shanghai, China). Endotoxin-free plasmids were prepared using an EndoFree plasmid purification kit (OMEGA, USA). Plasmids were adjusted to a final concentration of 1 mg/mL in PBS and stored at −20°C.

### 2.3. Animals and Immunization Protocol

4-5-week-old pathogen-free BALB/c male mice were purchased from the Laboratory of Animals Institute in Sichuan University (Chengdu, China). The vaccination schedules of mice are shown in Table 1. Groups of BALB/c (*n* = 18) were primed with PBST, BCG-China, or BCG-Pasteur1173 at week 0 and boosted with plasmid DNA or control plasmid at week 3 and week 6. Mice were immunized subcutaneously with 5∗10^6^ CFU of BCG in a volume of 0.1 mL per mouse and intramuscularly with 50 *μ*g DNA in a volume of 0.1 mL each time per mouse. Mice were put to death at 10, 14, 18, and 22 weeks (four mice for each group at each time point). Blood was collected from retroorbital sinus and the sera were stored at −20°C after separation until used.

### 2.4. ELISA for Antibody Response

Specific antibodies against TB were determined by an indirect ELISA method. The method was described as previous [22, 26]. Each sera sample was tested in three replicates, and the results are expressed as mean ± standard errors.

### 2.5. Proliferation of Splenocytes

The animals were sacrificed as previously described and the spleens were removed aseptically. The proliferation of lymphocyte were tested by MTT assay [3-(4,5-dimethylthiazol-2-yl)-2,5-diphenyl tetrazoliumbromide]. The method was described as previous [22, 27]. The results are expressed as the value of stimulation index (SI). SI = OD of stimulated well/OD of unstimulated well.

### 2.6. Flow Cytometry

The splenocytes were prepared and cultured as previously described [27, 28], and the splenocytes were plated in 6-well flat-bottom plates (5∗10^6^ cells in 2 mL of cRPMI per well) with 100 *μ*L TB-PPD (1 *μ*g/mL; XiangRui Biotech, Ltd., Beijing, China) in each well and incubated for 72 h (37°C, 5%CO_2_). The cells were collected and washed three times with 0.1 M PBS (PH = 7.2), and then rabbit anti-Mouse CD4^+^-PE and anti-Mouse CD8^+^-FITC (eBioscience, USA) were added into EP tube for a 30 min incubation in an ice-bath keep out of the sun. Finally, the cells were washed twice again and the proportions of CD4^+^ and CD8^+^ T cells were determined by flow cytometry (FACSCalibur, BD).

### 2.7. Cytokine Release Assay

The splenocytes were dealt with in the same way as flow cytometry assay previously described, and concentrations of IFN-*γ* and IL-4 in the medium were measured by an ELISA kit (eBioscience, USA) according to the manufacture's protocol.

### 2.8. Statistical Analysis

Measurements of these data are expressed as the mean ± standard errors (S.E.). We used one-way ANOVA to analyze the differences among the groups and post hoc test to analyze the differences between two groups. When *P*  value < 0.05, the differences were considered statistically significant.

## 3. Results

### 3.1. Antibodies in the Serum

**Figure 1:**
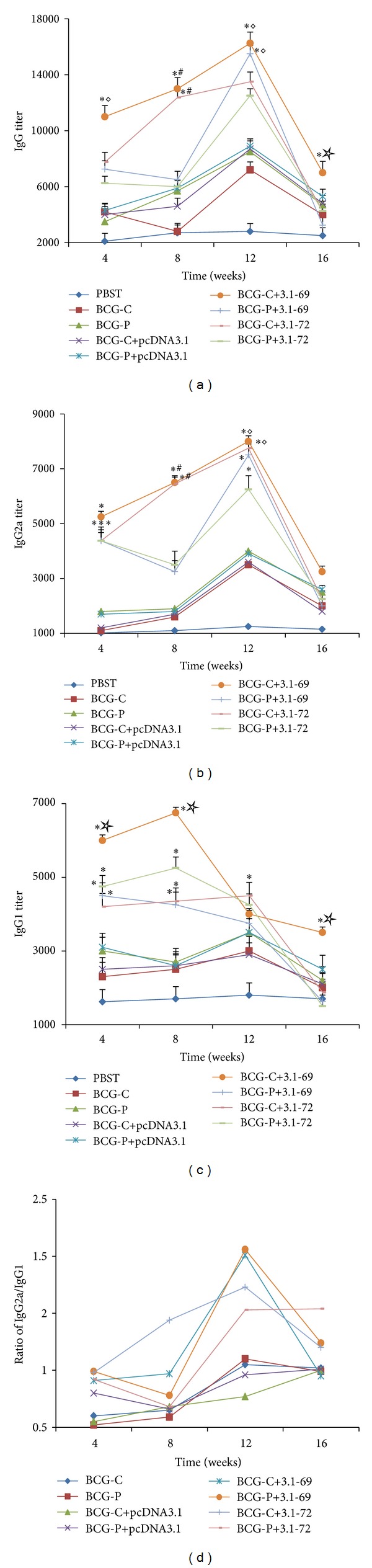
Analysis of the antibody responses via testing the IgG, IgG1, and IgG2a by ELISA. Animals were immunized and harvested at the indicated time points. The sera were obtained and tested for specific antibody levels. Results are expressed as mean ± standard errors. *P < 0.01 versus PBST, BCG-C, BCG-P, BCG-C+pcDNA3.1, or BCG-P+pcDNA3.1 groups. ^#^ *P* < 0.01 versus BCG-P+3.1-69 or BCG-P+3.1-72 group. ^◊^ *P* < 0.05 versus BCG-P+3.1-72 group. ^☆^ *P* < 0.05 versus BCG-P+3.1-69, BCG-C+3.1-72 or BCG-P+3.1-72 group.

The antibody titers were detected by ELISA assay to reflect the humoral immune response against TB. The levels of antibody response in the sera of the immunized mice at different time points are shown in Figure 1. The results show that firstly the titers of IgG and IgG2a antibodies in the group immunized with BCG-C+3.1-69 and BCG-C+3.1-72 were higher than those in the other 7 groups at the 8th week (*P* < 0.01); secondly the IgG titers in group BCG-C+3.1-69 were higher than those in the BCG-P+3.1-72, PBST, plasmid controls, and positive controls at the 4th and 12th weeks (*P* < 0.05) and were higher than those in the other 8 groups at the 16th week (*P* < 0.05); thirdly the titers of IgG2a antibodies in the group immunized with BCG-C+3.1-69 and BCG-C+3.1-72 were higher than those in the BCG-P+3.1-72, PBST, plasmid controls, and positive controls at the 12th week (*P* < 0.05). Besides, the titers of IgG1 antibodies in the group immunized with BCG-C+3.1-69 were higher than those in the other 8 groups at the 4th, 8th, and 16th weeks (*P* < 0.05).  Figure 1(d) shows that groups BCG-C+3.1-69, BCG-C+3.1-72, BCG-P+3.1-69, and BCG-P+3.1-72 all indicated a shift towards a Th1 immune response at the 12th week.

### 3.2. Lymphoproliferation Assay

**Figure 2:**
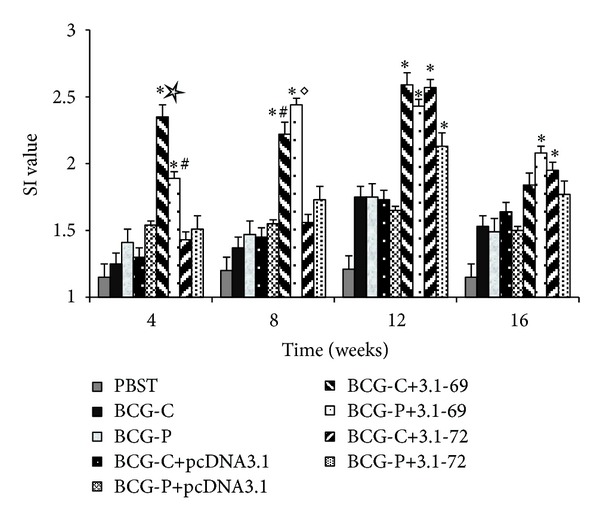
Analysis of the lymphoproliferative response to the prime-boost approach. Splenocytes were prepared from individual mice after the animals were killed, and proliferation was analyzed by MTT method using the TB-PPD stimulating as described in Section 2. The proliferation was measured by SI, and the data are presented as means ± standard errors. *P < 0.01 versus PBST, BCG-C, BCG-P, BCG-C+pcDNA3.1, or BCG-P+pcDNA3.1 groups. ^#^ *P* < 0.05 versus BCG-C+3.1-72 group. ^◊^ *P* < 0.05 versus BCG-C+3.1-72 or BCG-P+3.1-72 group. ^☆^ *P* < 0.05 versus BCG-P+3.1-69, BCG-C+3.1-72, or BCG-P+3.1-72 groups.

To detect the cell-mediated immune response, the splenic lymphocyte proliferation was assessed by MTT assay. The results show that the proliferation of splenocytes in the BCG-C+3.1-69, BCG-C+3.1-72, BCG-P+3.1-69, and BCG-P+3.1-72 groups were higher than those in the PBST, plasmid controls, and positive controls at the 12th week (*P* < 0.01), but there were no significative statistical differences between the above four groups (BCG-C+3.1-69, BCG-C+3.1-72, BCG-P+3.1-69, and BCG-P+3.1-72), while the group BCG-C+3.1-69 showed greater proliferation of splenocytes than the other 8 groups at the 4th week (*P* < 0.05) and than the BCG-C+3.1-72, PBST, plasmid controls, and positive controls groups at the 8th week (*P* < 0.05). The SI value in the BCG-C+3.1-69 and BCG-C+3.1-72 groups peaked at the 12th week, and the proliferation level decreased after this time. Importantly, the proliferation reaction of group BCG-C+3.1-72 still maintains at a high level at the 16th week (Figure 2).

### 3.3. Percentages of Splenocyte Subsets

**Figure 3:**
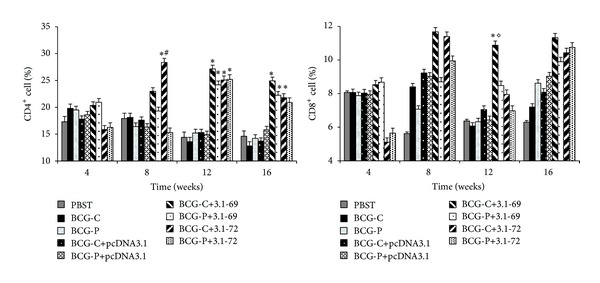
Analysis of the percentages of CD4^+^ and CD8^+^ cells (%). Animals were immunized and killed; the splenocytes were collected and handled as described in Section 2. Flow cytometry was used to determine the proportions of splenocyte subsets, and the results are presented as mean ± standard errors. *P < 0.01 versus PBST, BCG-C, BCG-P, BCG-C +pcDNA3.1, or BCG-P+pcDNA3.1 groups. ^#^ *P* < 0.05 versus BCG-P+3.1-69 or BCG-P+ 3.1-72 group. ^◊^ *P* < 0.05 versus BCG-P+3.1-69, BCG-C+3.1-72, or BCG-P+3.1-72 groups.

The proportions of splenocyte subsets were measured by flow cytometry. As Figure 3 shows, the BCG-C+3.1-72 group induced a significantly greater ratio of CD4^+^ T cells at the 8th week compared with BCG-P+3.1-69, BCG-P+3.1-72, PBST, plasmid controls, and positive controls groups (*P* < 0.05). In addition, the BCG-C+3.1-69, BCG-C+3.1-72, BCG-P+3.1-69, and BCG-P+3.1-72 groups induced a significantly greater ratio of CD4^+^ T cells at the 12th week compared with PBST, plasmid controls, and positive controls groups (*P* < 0.01), and the status of BCG-C+3.1-69, BCG-C+3.1-72, and BCG-P+3.1-69 groups lasted to the 16th week except the group BCG-P+3.1-72. Finally, the proportions of CD8^+^ T cells in the BCG-C+3.1-69 group was higher than that in the other 8 groups at the 12th week (*P* < 0.05) (Figure 3).

### 3.4. Cytokine Production

**Figure 4:**
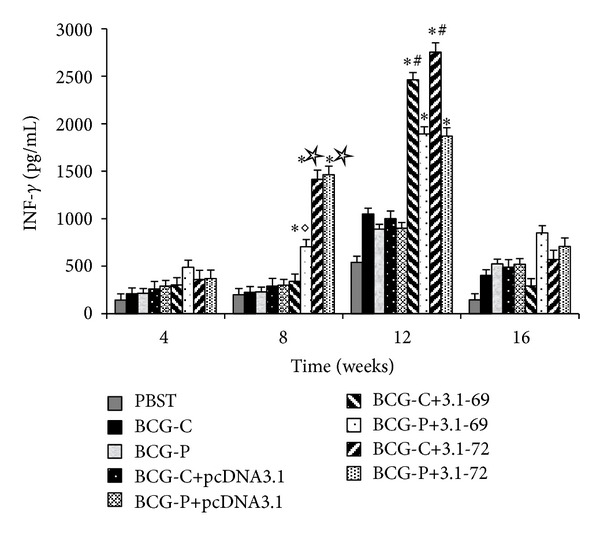
Analysis of IFN-*γ* released from splenocytes. After animals were harvested; splenocytes were collected and prepared as described in Section 2. IFN-*γ* concentration in the medium was measured by ELISA kit according to the manufacturer's protocol. Triplicate tests were done for each medium sample. *P < 0.01 versus PBST, BCG-C, BCG-P, BCG-C+pcDNA3.1, or BCG-P+pcDNA3.1 groups. ^#^ *P* < 0.05 versus BCG-P+3.1-69 or BCG-P+3.1-72 group. ^◊^ *P* < 0.05 versus BCG-C+3.1-69 group. ^☆^ *P* < 0.05 versus BCG-C+3.1-69 or BCG-P+3.1-69 group.

To determine Th1- and Th2-type immune response, the IFN-*γ* and IL-4 were detected from restimulated spleen cells by ELISA. According to Figure 4, the results clearly showed that the concentrations of IFN-*γ* in the group BCG-C+3.1-69 and BCG-C+3.1-72 were higher than those in the other 7 groups at the 12th week (*P* < 0.05), and the IFN-*γ* concentrations of the BCG-C+3.1-72 were higher than those of the groups BCG-C+3.1-69, BCG-P+3.1-69, PBST, plasmid controls, and positive controls at the 8th week (*P* < 0.05) (Figure 4). IL-4 maintained at a low level, and there were no significantly changes among any groups including PBST group (*P* > 0.05) (data not shown).

## 4. Discussion

Heterologous prime/boost vaccination strategies employing recombinant bacteria, viruses, proteins, and naked DNA have been shown to elicit stronger and more diverse cellular immune responses than BCG vaccine alone [5–7, 22]. In humans, DNA vaccines alone have not provided satisfactory results, whereas DNA vaccines produced better outcomes when immunized as a prime-boost strategy [29, 30]. According to these former observations, we built this work to evaluate the immunogenicity of two genes (Rv1769 and Rv1772) by a heterologous prime/boost strategy. The data of this paper supports the theory that heterologous prime-boost vaccination significantly induces more robust cellular immune responses than BCG vaccine alone.

Previously, numerous prime-boost vaccination protocols have demonstrated varying success when carried out in various infectious disease models. In tuberculosis experiments, prime-boost vaccination protocols include BCG/protein prime-boost [8, 22], DNA/protein prime-boost [31], DNA/adenovirus 5 prime-boost [32], DNA/BCG prime-boost [33], and BCG/MVA prime-boost [5] vaccination schedules, and these prime-boost protocols used Ag85A [5, 8], MT_1721 [31], ESAT-6 [33], Rv1769, and Rv1772 [22] as antigens. Considering a BCG immunization is done in early childhood, our study has begun with a BCG prime.

Antigens Rv1769 and Rv1772 are interesting because the coding genes are present in the genome of BCG-China, only deleted from the BCG-Pasteur. We suppose that overexpression of the genes enhances the immune response of the existing BCG. More importantly, we succeeded in improving the vaccine immunogenicity of the BCG by using a prime-boost vaccination.

The pathogenic bacterium of TB is* Mycobacterium tuberculosis*, which is intracellular bacteria and cell immune is very important in clearance of it. In the early infection, the CD4^+^ T cell can release IFN-*γ*, IL-2, and TNF-*α*, which can activate macrophages to fight against* M. tuberculosis* [34]. In this paper, IFN-*γ*, as the representative, was detected by ELISA, and the results show that groups BCG-C+3.1-69 and BCG-C+3.1-72 were of the highest levels of IFN-*γ* concentrations. In addition, flow cytometry results show that groups BCG-C+3.1-69 and BCG-C+3.1-72 can induce the greatest ratio of CD4^+^ T cells. According to the data of flow cytometry, we have observed that CD4^+^ T cells and IFN-*γ* levels of BCG-C+3.1-72 rise from 8th week and down after the 12th week; however, great ratio of CD4^+^ T cells of BCG-C+3.1-69 lasted to even the 16th week. So, we suppose that the group BCG-C+3.1-69 may enhance a stronger and longer-lasting T-cell immune response against* M. tuberculosis* early infection. Recently, numerous studies indicate that CD8^+^ T cells are critical for the induction of protective TB immunity in humans [35], NHPs [36], rodents [37], and cattle [38]. The CD8^+^ T cells can secrete perforin, granulysin, and extracellular enzyme to promote bacterial schizolysis [13, 34]. In our research, flow cytometry results show that the proportions of CD8^+^ T cells in the BCG-C+3.1-69 group was the highest at the 12th week with *P* < 0.05. Considering these, we have thought about the group BCG-C+3.1-69 not only induced polyfunctional CD4^+^ T cells, but also a robust CD8^+^ T-cell response in mice.

To measure the TH1-type immune response better, we also determined the antibody responses induced by these vaccine candidates. The results reveal that groups BCG-C+3.1-69 and BCG-C+3.1-72 can elicit high levels of IgG and IgG2a antibodies and also last a long time. Besides, the ratio of IgG2a/IgG1 shows a shift towards a Th1-type immune response.

Lastly, the proliferation rate of splenocytes increased consistently with the other experimental results; the splenocytes proliferation rate in group BCG-C + 3.1-69 rises in the 4th week and reaches the peak value in the 12th week. Due to the arguments above, we believe that the group BCG-C + 3.1-69 can induce stronger and longer-lasting TH1-type immune response than native BCG or other prime-boost groups in mice having CD4^+^ and CD8^+^ T cells.

Some research has demonstrated that BCG and recombinant BCG can induce central memory CD8^+^ T-cell differentiation* in vivo* [6, 39]. Because CD4^+^ T helper cells are important for driving memory CD8^+^ T-cell differentiation [40, 41], the ability of BCG to generate memory CD8^+^ T cells could make it have the propensity to stimulate CD4^+^ T helper cells growth. It has been suggested that the functional heterogeneity of T-cell responses may be associated with successful containment of microbial infections. The extent of T-cell polyfunctionality has been correlated with the protection against leishmaniasis in mice, HIV-1 in humans [42, 43], and SIV in nonhuman primates [44]. In this study, a prime BCG-boost DNA strategy can induce CD8^+^ T-cell differentiation as well as CD4^+^ T cell; we suppose that this vaccination strategy may obtain success against TB in humans and gene Rv1769 may be an excellent vaccine candidate.

The prime BCG-boost plasmid Rv1769 strategy improved the vaccine immunogenicity of BCG, and the subunit vaccination can be used to improve preexisting immunogenicity evoked by BCG and even be used in future clinical trials. More recent protocols show that naked DNA performs better in macaques and humans as part of a prime-boost regime [30]. Therefore, BCG prime and naked DNA boost vaccination should be used for several reasons: firstly BCG needs to be included in future vaccine trials against tuberculosis; secondly naked DNA vaccination has been shown to be effective as a submit vaccine in prime-boost vaccination protocols; lastly this vaccination regime can improve BCG-induced immunogenicity.

In summary, our results provide evidence that a BCG-naked DNA prime-boost vaccination protocol represents a valuable candidate (gene Rv1769) for future vaccine trials targeted at one of the major health problems worldwide.

In the future, we would consider building an* in vivo* challenge model to extend our findings to an infection/disease protection system.

## Figures and Tables

**Table 1:** Heterologous prime-boost immunization schedule.

Group	Prime	Boost1	Boost2
PBST	PBST	PBST	PBST
BCG-C	BCG-China	PBST	PBST
BCG-P	BCG-Pasteur1173	PBST	PBST
BCG-C+pcDNA3.1	BCG-China	Plasmid pcDNA3.1	Plasmid pcDNA3.1
BCG-P+pcDNA3.1	BCG-Pasteur1173	Plasmid pcDNA3.1	Plasmid pcDNA3.1
BCG-C+3.1-69	BCG-China	Plasmid pc-Rv1769	Plasmid Pc-Rv1769
BCG-P+3.1-69	BCG-Pasteur1173	Plasmid pc-Rv1769	Plasmid Pc-Rv1769
BCG-C+3.1-72	BCG-China	Plasmid pc-Rv1772	Plasmid Pc-Rv1772
BCG-P+3.1-72	BCG-Pasteur1173	Plasmid pc-Rv1772	Plasmid Pc-Rv1772
Timeline in weeks	0 ↓	3 ↓	6 ↓

## Abbreviations

PBST: PBS-Tween 80
BCG: Bacille Calmette-Guérin
BCC-C: The group immunized with vaccine BCG-China substrain
BCG-P: The group immunized with vaccine BCG-Pasteur1173 substrain
BCG-C + pcDNA3.1: The group immunized BCG-China prime plasmid pcDNA3.1 boost
BCG-P+pcDNA3.1: The group immunized BCG-Pasteur1173 prime plasmid pcDNA3.1 boost
BCG-C+3.1-69: The group immunized BCG-China prime plasmid pcDNA3.1-Rv1769 boost
BCG-P+3.1-69: The group immunized BCG-Pasteur1173 prime plasmid pcDNA3.1-Rv1769 boost
BCG-C+3.1-72: The group immunized BCG-China prime plasmid pcDNA3.1-Rv1772 boost
BCG-P+3.1-72: The group immunized BCG-Pasteur1173 prime plasmid pcDNA3.1-Rv1772 boost.
